# First Report of IMI-2-Producing *Enterobacter bugandensis* and CTX-M-55-Producing *Escherichia coli* isolated from Healthy Volunteers in Tunisia

**DOI:** 10.3390/antibiotics12010116

**Published:** 2023-01-08

**Authors:** Rym Ben Sallem, Ameni Arfaoui, Afef Najjari, Isabel Carvalho, Abdelmalek Lekired, Hadda-Imen Ouzari, Karim Ben Slama, Alex Wong, Carmen Torres, Naouel Klibi

**Affiliations:** 1 Laboratory of Microorganisms and Active Biomolecules (LR03ES03), Faculty of Sciences of Tunis, University of Tunis El Manar, Tunis 2092, Tunisia; 2 Bioresources, Environment and Biotechnology Laboratory (LR22ES04), Higher Institute of Applied Biological Sciences of Tunis, University of Tunis El Manar, Tunis 1006, Tunisia; 3 Department of Sciences, Sainte Anne University, 1695 Route 1, Clare, NS B0W 1M0, Canada; 4 Biochemistry and Molecular Biology, University of La Rioja, 26006 Logrono, Spain; 5 Department of Veterinary Sciences, University of Trás-os-Montes-and Alto Douro, 5000-801 Vila Real, Portugal; 6 Department of Biology, Carleton University, 1125 Colonel by Drive, Ottawa, ON K1S 5B6, Canada

**Keywords:** ESBL, carbapenemases, *E. bugandensis*, IMI-2, healthy humans, Tunisia

## Abstract

The aim of this study was to characterize the prevalence of fecal carriage of extended-spectrum beta-lactamases and carbapenemase-producing Gram-negative bacteria among healthy humans in Tunisia. Fifty-one rectal swabs of healthy volunteers were plated on MacConkey agar plates supplemented with cefotaxime or imipenem. The occurrences of resistance genes, integrons, and phylogroup typing were investigated using PCR and sequencing. The genetic relatedness of isolates was determined by pulsed-field-gel-electrophoresis (PFGE) and multilocus-sequence-typing (MLST). Whole-genome-sequencing (WGS) was performed for the carbapenem-resistant isolate. Sixteen ESBL-producing *Escherichia coli* isolates and one carbapenem-resistant *Enterobacter bugandensis* were detected out of the fifty-one fecal samples. The ESBL-producing *E. coli* strains contained genes encoding CTX-M-15 (n = 9), CTX-M-1 (n = 3), CTX-M-27 (n = 3), and CTX-M-55 (n = 1). Three CTX-M-1-producers were of lineages ST131, ST7366, and ST1158; two CTX-M-15-producers belonged to lineage ST925 and ST5100; one CTX-M-27-producer belonged to ST2887, and one CTX-M-15-producer belonged to ST744. Six isolates contained class 1 integrons with the following four gene cassette arrangements: *dfrA5* (two isolates), *dfrA12-orf-aadA2* (two isolates), *dfrA17-aadA5* (one isolate), and *aadA1* (one isolate). *E. bugandensis* belonged to ST1095, produced IMI-2 carbapenemase, and contained *qnrE1* and *fosA* genes. A genome-sequence analysis of the *E. bugandensis* strain revealed new mutations in the *bla*_ACT_ and *qnr* genes. Our results reveal an alarming rate of ESBL-*E. coli* in healthy humans in Tunisia and the first description of IMI-2 in *E. bugandensis.*

## 1. Introduction

Antimicrobial resistance is observed in both pathogenic and normal commensal bacteria. The bacterial microbiota contains various antimicrobial resistance genes (ARGs), even in individuals with no history of exposure to antibiotics [1]. Antimicrobial-resistant bacteria in the gut microbiota of humans and animals may pose a serious threat as they can spread to other hosts or transfer genetic resistance elements to other members of the microbiota, including pathogens [2,3].

*Enterobacteriaceae* are a large family of Gram-negative bacteria that includes bacteria of the normal gut microbiota but also a number of pathogens for humans. They are a common cause of urinary-tract infections and hospital-acquired pneumonia. Beta-lactam resistance in these pathogens is most commonly due to the expression of beta-lactamases, including extended-spectrum beta-lactamases (ESBLs) and carbapenemases. ESBLs mediate resistance to all penicillins, expanded-spectrum cephalosporins (ESCs) (e.g., ceftazidime, cefotaxime, and ceftriaxone), and aztreonam, but not to cephamycins (cefoxitin and cefotetan) and carbapenems. Carbapenemases confer resistance to penicillins and carbapenems, which are the drug of choice for the treatment of severe infections caused by ESBL-producing *Enterobacteriacae* (ESBL-E), and to ESCs used in the treatment of some of those infections (KPC, NDM, OXA, VIM, etc.). To date, many different types of ESBLs and carbapenemases have emerged worldwide (http://www.bldb.eu) accessed on 1 November 2022 [4].

Carbapenem- and ESC- resistant *Enterobacteriaceae* are rising on the African continent, reaching alarming levels in some countries. In a recent review on carbapenemase-producing *Enterobacteriaceae* (CPE) in Africa, their prevalence in hospital settings was reported as ranging from 2.3% to 67.7% in North Africa and from 9% to 60% in sub-Saharan Africa [5]. Moreover, the Mediterranean and North African countries have recently been designated as CPE-endemic areas, where ESBL-E and carbapenem-resistant *Enterobacteriaceae* (CRE) have been frequently reported from clinical, non-clinical, and environmental sources [5,6]. However, there is still limited information regarding the prevalence and characteristics of CPEs and ESBL-E among healthy humans in African countries. Data on the prevalence of ESBL-E in the community remain scarce in Tunisia. While the rate of fecal carriage of ESBL-E has mainly been investigated during nosocomial outbreaks [7], only one study was conducted in 2011 in a community setting. This study reported a low fecal carriage rate (7.3%) of ESBL-E among healthy individuals [8]. In the current report, we aimed to assess the prevalence of ESBL-Es and CREs among healthy humans in Tunisia in order to determine the beta-lactam resistance genes and to characterize their clonal relatedness.

## 2. Results

### 2.1. The Fecal Carriage Rate of Detected ESBL and Carbapenemase-Producing Enterobacteriaceae and Beta-Lactamases

Sixteen (31.4%) out of fifty-one fecal samples yielded ESBL-E, while CPE was identified in only one sample. All ESBL-E were *E. coli* isolates. The prevalence of different ESBL types among the *E. coli* isolates is shown in Table 1. ESBL-*E. coli* all possessed a CTX-M-type ESBL: *bla*_CTXM-15_ (n = 9), *bla*_CTX-M-1_ (n = 3), *bla*_CTX-M-27_ (n = 3) and *bla*_CTX-M-55_ genes (n = 1). Three of the CTX-M-positive isolates were also positive for *bla*_TEM-1_.

### 2.2. Phenotypic and Genotypic Antimicrobial-Resistance Patterns of ESBL-Producing E.coli Isolates

All *E. coli* ESBL-producing isolates were found to be resistant to ampicillin, ceftazidime, cefotaxime, and sulphonamides. Additionally, high resistance rates were observed for tetracycline (81.5%), streptomycin (81.25%), sulphamethoxazole-trimethoprim (68.75%), and nalidixic acid (43.3%). In contrast, amikacin, kanamycin, tobramycin, and gentamicin were the most effective antibiotics, with susceptibility rates of 93.75%, 87.5%, 87.5%, and 75%, respectively (Table 2).

Six ESBL-positive isolates contained class 1 integrons with the following four gene cassette arrangements: *dfrA5* (two isolates), *dfrA12-orf-aadA2* (two isolates), *dfrA17-aadA5* (one isolate), and *aadA1* (one isolate).

A variety of resistance genes located outside integrons were observed among our strains: *tet(A)* or *tet(B)* (in 12 tetracycline-resistant strains), and *sul2*, *sul3* (in the 16 sulphonamide-resistant strains). 

### 2.3. Molecular Typing of Isolates

Phylogenetic analysis of *E. coli* isolates revealed that most ESBL isolates (9/16; 56.3%) were classified within phylogenetic group A, six (37.5%) were members of group D, and one (6.2%) was a member of group B2. The PFGE analysis showed that all of the isolates were genetically unrelated (supplementary Figure S1). MLSTs were determined for seven selected ESBL-*E. coli* depending on the type of CTX-M produced. ST131, ST7366, and ST1158 were detected in three *bla*_CTX-M-1_-containing isolates; ST925, and ST5100 were found in two *bla*_CTX-M-15_-containing isolates; ST2887 in one *bla*_CTX-M-27_-producing isolate; and ST744 in one *bla*_CTX-M-55_-producing isolate (Table 2).

### 2.4. Genotypic and Phenotypic Characterization of the Carbapenem-Resistant Isolate

Genotypic and phenotypic identification showed that the carbapenem-resistant isolate, the 9i strain, belonged to the species *Enterobacter bugandensis*. PCR and sequencing revealed the presence of *bla*_IMI-2_. This strain demonstrated multidrug resistance to all tested antibiotics except colistin. A whole-genome-based phylogenetic tree created using TYGS illustrated that strain 9i is close to the *E. bugandensis* EB-247 (NZ_AP022508.1) species (supplementary Figure S2). The ANI values of strain 9i with all genomic sequences of *E. bugandensis* strains deposited in GenBank (since 2022) range from 85.65% for *E. bugandensis* (GCF 002890755.1) to 95.23% for *E. bugandensis* strain 153_ECLO (NZ_JVSD00000000.1) (supplementary Table S5). These results, indicating an ANI value above the 95% cutoff for the same species, demonstrate that the 9i strain is related to the *E. bugandensis* species.

*E. bugandensis* 9i (NZ_JAMYWA000000000.1) consists of 5.12 Mbp, distributed over 103 contigs and organized in 67 scaffolds with a fold coverage of 80×, having an N50 contig size of 221,597 kb and an average G + C content of 56%. Mining the 9i genome sequence revealed the *qnrE1* and *fosA* genes encoding resistance to fluoroquinolone and fosfomycin, respectively. Interestingly, a novel variant of the ACT AmpC beta-lactamase encoding gene (Locus tag_M6B24_02185) was identified. In fact, the AmpC gene encoded a protein (MCP1112056.1) that showed 99% similarity with the beta-lactamase genes coding for ACT-77 (WP_060573544.1). The AmpC sequence identified in our study includes SNPs encoding four amino-acid substitutions at positions 35 (H/N), 115 (E/D), P 203 (R/K), and 267 (P/S) of the amino-acid chain (supplementary Table S1). On the other hand, we detected a new variant of the *qnrE* gene with a 99.38% similarity in identity to QnrE3 (CP014280.1) compared to the Qnr proteins available in the databases; and three amino-acid alterations were observed in the current variant (G7S), (A94D) and (A99V). The amino-acid alterations (G7S) and (A99V) were detected in previously published Qnr family proteins under accession numbers WP_218307411.1 and WP_039992288.1, respectively (supplementary Table S2), while the alteration at position 94 is new.

## 3. Discussion

This study was conducted to determine the prevalence of ESBLs and CPEs among *Enterobacteriaceae* isolates recovered from the feces of healthy individuals in Tunisia. Our findings showed that 31.4% of volunteers were ESBL-E carriers. In addition, one healthy volunteer was colonized by an IMI-2-producing *E. bugandensis*; to the best of our knowledge, this is the first detection of an asymptomatic CPE carrier, not only in Tunisia but also in North Africa.

The prevalence of fecal ESBL-E found in our study is almost four times higher than it was when we reported on it nine years earlier [8]. Comparing these two studies, we found that the increased prevalence of fecal ESBL-E is associated with a change in CTX-M-type distribution; indeed, CTX-M-1 was the only enzyme responsible for the ESBL phenotype in the population studied in 2011, while today more CTX-M alleles, including CTX-M-27, 55 and 15, are present in the digestive tract of the Tunisian population analyzed.

The dissemination of ESBLs among the community confirms that they are not confined solely to the clinical environment. The variability and proportion of ESBLs detected in this study seem to be in agreement with the current epidemiological data on the spread of ESBL *E. coli* in Tunisia. Indeed, our results showed that the most frequent ESBL among human *E. coli* samples is CTX-M-15, which was found in nine samples. This enzyme was also detected in ESBL-producing isolates obtained from the hospital environment, wastewater, food, and animals [9,10,11]. On the other hand, CTX-M-1 constitutes the most common ESBL of animal origin, and it is also frequent in the natural environment and in food [2,12,13,14].

CTX-M-27-producing *E. coli* isolates are less common and have been recently reported in municipal wastewater [11]. Interestingly, the *bla*_CTX-M-55_ gene found in one ESBL-*E. coli* has never been reported in Tunisia in human isolates, and only in a few *E. coli* isolates of animal origin [14].

The reported rate of fecal ESBL-E carriage in our study is comparable to the rates reported in Turkey and in Tanzanian communities (34.4%) and to the rate reported for healthy children living in a rural village in Venezuela (34.6%) [15,16,17]. By contrast, occurrences in Egypt (21%), India (19%), Spain (16%), the Netherlands (15.9%), Japan (15.6%), Saudi Arabia (12.3%), Kuwait, (13.2%), Qatar (9%), Libya, and Sweden (4.7%) are lower, suggesting possible variations in the policies adopted by different countries for the prevention of the spread of antimicrobial resistance [18,19,20,21,22,23,24,25,26,27].

The high fecal colonization of ESBL and Carbapenemase-producing Enterobacteriaceae was attributed to the effects of different risk factors. Although their origins and characteristics were not investigated in our study, possible reasons for the acquisition of resistance could be the extensive utilization of self-administered antibiotics in the Tunisian community and the consumption of animal products harboring resistant bacteria [2].

A study carried out by Kothari et al., wherein gut colonization of exclusively breastfed healthy neonates was investigated, showed widespread resistance to ampicillin (87%) and cephalosporins among Enterobacteriaceae. The authors hypothesized that the acquisition of resistance genes was achieved through breastfeeding and contact with pets, as well as through horizontal transfer in the gut microbiota [28]. Likewise, in a Swedish study, tetracycline resistance was detected in 12% of commensal *E. coli* strains from infants who had not received tetracycline [29]. Furthermore, two studies from Lebanon addressed the risk factors for ESBL carriage in nursing-home residents in Beirut and Tripoli. In Beirut, constipation and antibiotic intake were independent risk factors for ESBL carriage, whereas in Tripoli, only antibiotic administration was found [30,31].

PFGE analyses and MLST typing revealed high genetic diversity among ESBL-*E. coli* isolates. These isolates harbored different *bla*_CTX-M_ alleles and belonged to different non-epidemic *E. coli* sequence types, suggesting that commensal *E. coli* acts as a reservoir of *bla*_CTX-M_ genes. Through horizontal gene transfer, these *bla*_CTX-M_ genes may spread in the community amongst different bacterial species and strains. Their dissemination is often associated with highly mobilizable gene platforms, including plasmids and transposons; *bla*_CTX-M_ gene transfer is largely promoted by plasmids, which are often self-conjugative and carry additional resistance determinants [2]. Transposable elements carried by the plasmid can further mobilize the genes by copying them onto another plasmid or chromosome and so increase their potential for transmission to additional bacterial recipients through conjugation [2,8]. 

It is of note that the pandemic lineage of extraintestinal pathogenic *E. coli* ST131, known to cause urinary and bloodstream infections, was also observed. Our data are consistent with those obtained in other recent studies in the Netherlands and California, where the ESBL-*E. coli* ST131 strain was detected in the intestine of healthy subjects [32,33].

Interestingly, reduced susceptibility to imipenem was noted in only one strain isolated from a female volunteer. PCR and sequencing results confirmed by NGS analysis data showed that it was an IMI-2-producing *E. bugandensis.* This is a newly described species that was initially found during a neonatal outbreak in Tanzania [34]. Until now, only three studies have reported *E. bugandensis* from human and non-human origin, including the International Space Station, cattle vermicompost, and clinical samples [35,36,37]. Despite the fact that IMI carbapenemases are mainly found in *Enterobacter* spp., no study has reported the presence of such enzymes among *E. bugandensis*, with the exception of a recent study where the already published *E. cloacae* genome MBRL 1077, originally reported as an IMI-1-producing *E. cloacae* strain isolated in the USA, was reclassified as *E. bugandensis* [36,37]. Here, we report the first recognition of IMI-2-producing *E. bugandensis* isolated from healthy humans.

## 4. Materials and Methods

### 4.1. Isolation and Identification

Fifty-one rectal swabs were collected during the period from May 2018 to September 2018 from healthy human volunteers (with an age range of 3 months to 55 years) living in different urban areas in Tunisia. We excluded subjects who had undergone any antibiotic therapy or hospitalization in the 3 months prior to fecal sampling.

The samples were seeded on two MacConkey agar plates supplemented with cefotaxime (2 μg/mL) and imipenem (1 μg/mL), respectively, and were incubated at 37 °C for 24 h. For samples showing growth on the selective medium, at least three colonies were selected for subsequent characterization. Resistant isolates were identified using the VITEK 2 Compact system (bioMérieux, Marcy-l’Étoile, France) and by species-specific polymerase chain reaction (PCR) (amplification of the *uidA* gene) [8]. Only one ESBL or carbapenemase-positive isolate per sample was studied further.

#### 4.1.1. Antibiotic Susceptibility Tests

Antimicrobial susceptibility testing was carried out using the disk-diffusion method in accordance with the CLSI recommendations [38]. The antibiotics tested were as follows: ampicillin, cefoxitin, ceftazidime, cefotaxime, Amoxicillin-clavulanic acid, imipenem, ertapenem, meropenem, aztreonam, gentamicin, amikacin, tobramycin, streptomycin, nalidixic acid, ciprofloxacin, sulphonamides, colistin, trimethoprim–sulfamethoxazole, tetracycline, and chloramphenicol. Minimum inhibitory concentrations (MICs) of selected anti-microbial agents, namely colistin, imipenem, and ertapenem, were determined by broth microdilution in 96-well plates (Thermo Fisher Scientific, Waltham, MA, USA). *E. coli* ATCC 25922 was used as a control strain.

#### 4.1.2. Genetic Typing of Isolates

The clonal relationship among ESBL-producing *E. coli* isolates was determined with pulsed field gel electrophoresis (PFGE) using the XbaI enzyme, as previously described [39]. Seven selected ESBL-producing *E. coli* isolates were also characterized by multilocus sequence typing (MLST) in order to determine the corresponding sequence type (ST) and clonal complex (CC). Nucleotide sequences of the housekeeping genes were submitted to the *Escherichia coli* MLST Database: (http://mlst.warwick.ac.uk/mlst/dbs/Ecoli) [40]. The isolates were assigned to the phylogenetic groups A, B1, B2, or D using a PCR strategy with specific primers for *chuA*, *yjaA*, and TspE4.C2 determinants [41].

### 4.2. Detection and Characterization of Beta-Lactamase Genes and Other Resistance Genes

Plasmid-encoded AmpC (CMY, DHA, ACT), and ESBL (CTX-M, TEM, OXA, SHV) genes were analyzed using PCR and subsequent sequencing (supplementary Table S3) [42,43]. The presence of carbapenemase-encoding genes of class B (*bla*_IMP_, *bla*_VIM_, *bla*_NDM_, *bla*_GIM_, *bla*_SPM_, *bla*_SIM_), class A (*bla*_NMC_, *bla*_IMI_, *bla*_SME_, *bla*_KPC_, *bla*_GES_), and class D families (*bla*_OXA-48,_ *bla*_OXA-23_) was confirmed by performing PCR and sequencing, using previously described primers (supplementary Table S3) [44,45].

The presence of genes associated with resistance to tetracycline [*tet(A)* and *tet(B*)], sulphonamides [*sul1*, *sul2*, and *sul3*], gentamicin [*aac(3)-II* and *aac(3)-IV*], streptomycin [*strA* and *strB*], and quinolones [*qnr*, *qepA* and *aac(6′)-Ib-cr*] was investigated using PCR (supplementary Table S4) [39]. Detection and characterization of class 1 and 2 integrons and their variable regions were examined by PCR and sequencing, as previously described in [39]. The presence of *qacEΔ1*-*sul1* genes in the 3′-conserved segment of class 1 integrons was also investigated by PCR in all intI1-positive isolates [39].

### 4.3. Whole-Genome Sequencing (WGS), Assembly, and Annotation of E. bugandensis 9i

Genomic DNA was extracted using the QIAamp DNA Mini Kit according to the manufacturer’s recommendations. Whole-genome sequencing (WGS) was carried out on a MiSeq machine (Illumina Inc., San Diego, CA, USA). Quality assessment of the reads was performed using the FASTQC quality control tool version 0.10.0 [46]. Quality filtering was performed with the Trimmomatic program [47]. The reads assembling was performed by first using the Velvet de novo assembler for short read set assemblies [48]. Next, the results were reassembled with SPAdes 3.11.1 using multiple values for k-mer size (here, 33, 55, and 91) in order to enhance the quality of reads assemblies [49]. DNA contamination and confidence estimation for single-cell genome data were checked using ACDC software 2021–2022 [50]. Genome annotation was performed with NCBI Pro-karyotic Genome Annotation Pipeline (PGAP) [51]. This Whole-Genome Shotgun project has been deposited at DDBJ/ENA/GenBank under the accession no JAMYWA000000000. The version described in this paper is version JAMYWA010000000. 

Whole-genome-based taxonomic analysis of *E. bugandensis* 9i was then performed using the Type Strain Genome Server (TYGS) (at https://tygs.dsmz.De) [52]. The phylogenomic tree was constructed using FastME from the Genome BLAST Distance Phylogeny (GBDP) [53]. Then, pair-wise-genome comparisons were conducted using GBDP and inter-genomic distances which were obtained with algorithm ‘trimming’ and distance formula d5 [52]. Finally, trees were rooted at the midpoint and branch supports were inferred from 100 pseudo-bootstrap replicates [54].

The comparative genomic analysis was based on the average nucleotide identity using both best hits (one-way ANI) and reciprocal best hits (two-way ANI) between the genome sequence and all genome assemblies of *Enterobacter bugandensis* species (n = 172) available in GenBank (as of December 2022) using the ANI calculator with the program JspeciesWS [55]. Typically, the ANI values between genomes of the same species are above 95% [56].

## 5. Conclusions

Several ESBL-*E. coli* and imipenem-resistant *E. bugandensis* were found among healthy populations with a high fecal carriage rate. To the best of our knowledge, this is the first study detecting the fecal carriage of IMI-2-producing *E. bugandensis* in healthy populations in Africa and worldwide. However, due to the small sample size in our study, further studies are required to provide more compelling evidence in the near future. 

## Figures and Tables

**Table 1:** Demographic characteristics and prevalence of ESBL-E carriage in the study population.

Variable		Healthy Volunteers	ESBL-E Carriers
**Sex**	Men n (%)	22 (43.2 %)	7 (31.8 %)
	Women n (%)	29 (56.8%)	9 (31%)
**Age group**	3 m–10 y n (%)	12 (23.53%)	3 (25 %)
	11 y–20 y n (%)	16 (31.37%)	5 (31.2 %)
	21 y–55 y n (%)	23 (45.1 %)	8 (34.8 %)
**Total**		51	16

m: month, y: year.

**Table 2:** Characteristics of the 16 ESBL-producing *E. coli* isolates recovered from the fecal samples of healthy humans.

Strain	Resistance Profile to Non Beta-Lactams	Beta-Lactamases	MLST	Phylogroup	PFGE	Class 1 Integron		Other Resistance Genes Detected Outside Integron
						IntI1/ qacEΔ1 + sul1	Gene Cassette Arrangement	
3i	CIP, NAL, SXT, SUL, S, TET	CTX-M-15	ND	A	P1	+/+	aadA1	sul2, sul3, tetA
5c	CIP, NAL, SUL, TET	CTX-M-1	ST131	B2	P2	−/−	-	sul2, tetA
13c	NAL, SUL, S, GN, CHL	CTX-M-1	ST1158	D	P3	−/−	-	sul2
19c	SXT, SUL, S, TET	CTX-M-15	ST925	D	P4	−/−	-	sul2, tetA
25c	CIP, NAL, SXT, SUL, AK, K, T, S, GN, CHL, TET	CTX-M-15, TEM-1	ST5100	A	P5	+/+	dfrA12-orf-aadA2	sul1, sul2, tetB
26c	SUL, TET	CTX-M-15	ND	A	P6	−/−	-	-
30c	SXT, SUL, S, TET	CTX-M-27	ST2887	D	P7	−/−	-	sul1, sul2, tetA
32c	SXT, SUL, S, RAM, CHL	CTX-M-15	ND	A	P8	−/−	-	sul3
35c	SXT, SUL, S, TET	CTX-M-27	ND	D	P9	−/−	-	sul2, sul3, tetA
37c	SXT, SUL	CTX-M-15	ND	A	P10	+/+	dfrA5	sul2, tetA
39c	SXT, SUL, S, TET	CTX-M-15	ND	A	P11	−/−	-	sul2, tetA
42c	SXT, SUL, S, TET	CTX-M-15, TEM-1	ND	A	P12	+/+	dfrA12-orf-aadA2	sul2, tetA
45c	SUL, K, S, TET	CTX-M-27	ND	D	P13	−/−	-	sul2, tetA
46c	CIP, NAL, SXT, SUL, K, T, S, GN, CHL, TET	CTX-M-55	ST744	D	P14	+/+	dfrA17-aadA5	sul2, tetA
47c	CIP, NAL, SXT, SUL, S, TET	CTX-M-15	ND	A	P15	−/−	-	sul2
49c	CIP, NAL, SXT, SUL, S, TET	CTX-M-1, TEM1	St7366	A	P16	+/+	dfrA5	sul2, tetA

SXT: trimethoprim–sulfamethoxazole; SUL: sulphonamides; AK: amikacine; K: kanamycine; T: tobramycine, S: streptomycin, GN: gentamicin, TET: tetracycline; CHL: chloramphenicol; NAL: nalidixic acid; CIP: ciprofloxacin; ND: not done.

## Data Availability

Not applicable.

## Supplementary Materials

The following supporting information can be downloaded at: https://www.mdpi.com/article/10.3390/antibiotics12010116/s1. Table S1: Analysis of mutations in ACTi gene after comparison with ACT-77 (WP_060573544.1); Table S2: Analysis of mutations in qnr gene in *E. bugandensis* 9i genome sequence; Table S3: Primers of the target β-lactamase genes; Table S4: Primers of the target antimicrobial resistance genes; Table S5: ANI values of *E. bugandensis* 9i isolate with all genome sequences of *E. bugandensis* deposited in genbank (as of December 2022) Figure S1: Pulsed-field gel electrophoresis (PFGE) profiles of the ESBL-producing *E. coli* isolates recovered from the faecal samples of healthy humans; Figure S2: Phylogenomic tree based on genome sequences in the TYGS tree inferred with FastME 2.1.6.1 from Genome BLAST Distance Phylogeny approach (GBDP). The branch lengths are scaled in terms of GBDP distance formula d5. The numbers above branches are GBDP pseudobootstrap support values > 50% from 100 replications. The tree was rooted at the midpoint.
