# Supplementary material for: First Report of IMI-2-Producing Enterobacter bugandensis and CTX-M-55-Producing Escherichia coli isolated from Healthy Volunteers in Tunisia
Source: Antibiotics (Basel). 2023 Jan 8;12(1):116. doi: 10.3390/antibiotics12010116 (PMC9854954; doi:10.3390/antibiotics12010116)
Supplement: Supplementary file 1 [file antibiotics-12-00116-s001.zip › antibiotics-2095283-figures.pdf]

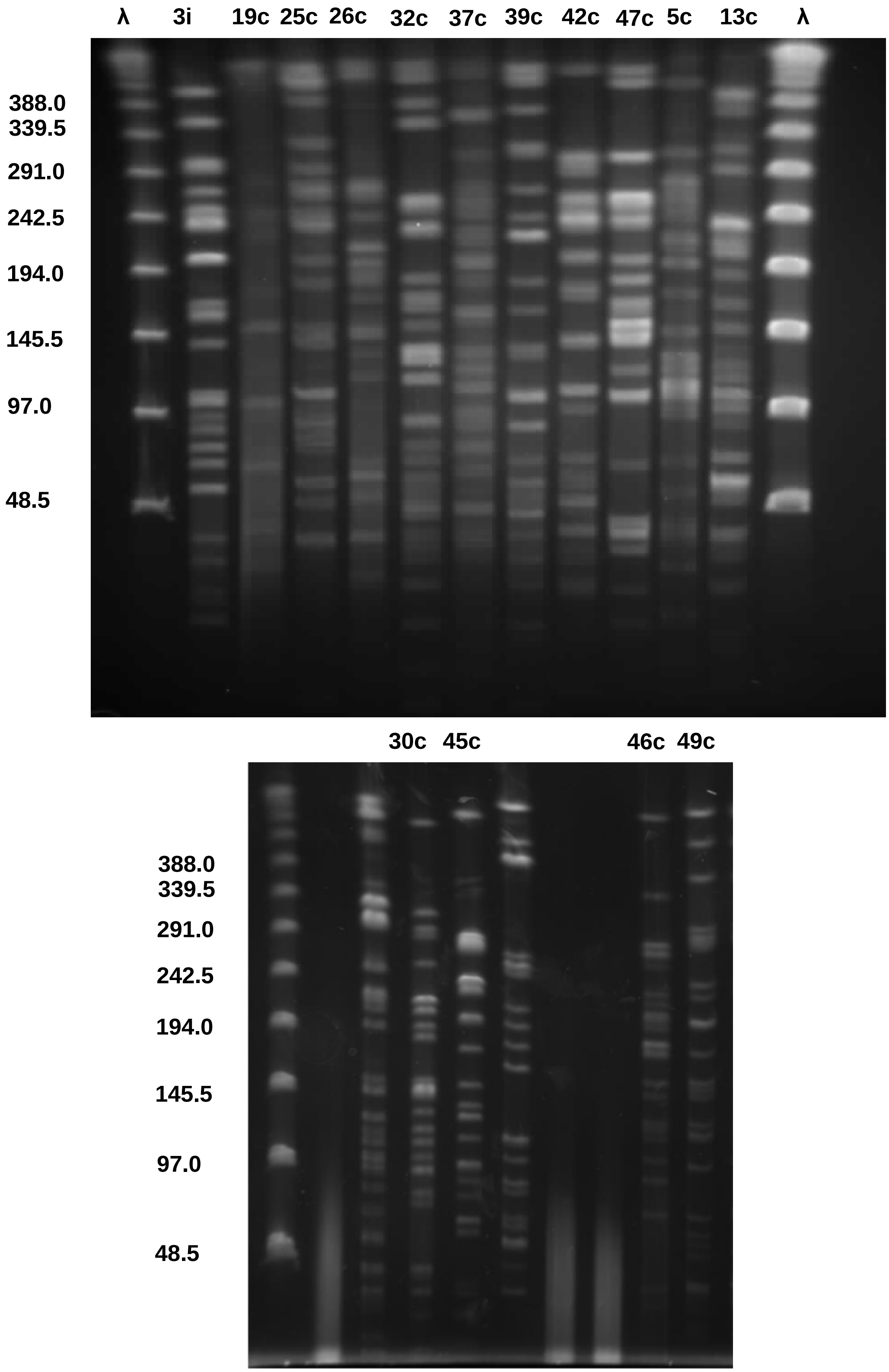

Supplementary Figure S1. Pulsed-field gel electrophoresis (PFGE) profiles of the ESBL-producing *E. coli* isolates recovered from the faecal samples of healthy humans

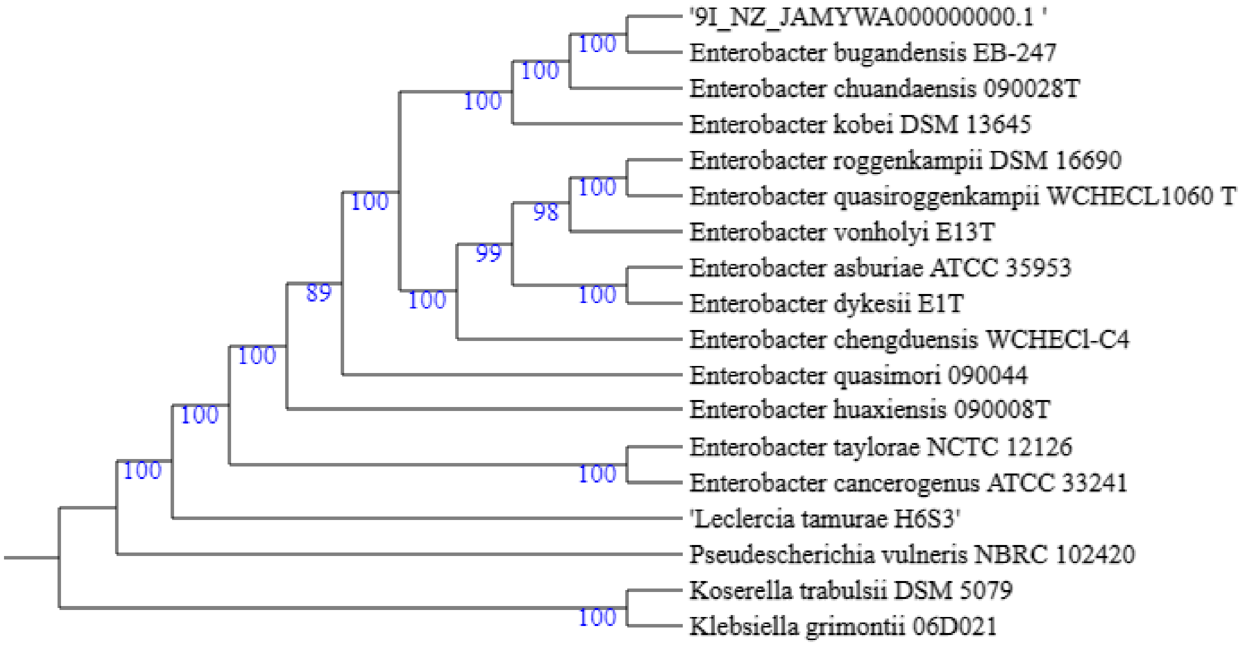

Supplementary Figure S2. Phylogenomic tree based on genome sequences in the TYGS tree inferred with FastME 2.1.6.1 from Genome BLAST Distance Phylogeny approach (GBDP). The branch lengths are scaled in terms of GBDP distance formula d5. The numbers above branches are GBDP pseudo-bootstrap support values>50% from 100 replications. The tree was rooted at the midpoint
