# Supplementary material for: First Report of IMI-2-Producing Enterobacter bugandensis and CTX-M-55-Producing Escherichia coli isolated from Healthy Volunteers in Tunisia
Source: Antibiotics (Basel). 2023 Jan 8;12(1):116. doi: 10.3390/antibiotics12010116 (PMC9854954; doi:10.3390/antibiotics12010116)
Supplement: Supplementary file 1 [file antibiotics-12-00116-s001.zip › antibiotics-2095283-supplementary tables.pdf]

Table S1 : Analysis of mutations in ACTi gene after comparison with ACT-77 (WP_060573544.1)

Mismatchs and positions	Position	Accession number of similar proteins with similar mismatches
H-N	P35	WP_061099492.1
E-D	P115	WP_192986658.1<br>WP_061099492.1<br>WP_164461280.1<br>WP_059354427.1
R-K	P 203	WP_061099492.1
P-S	P 267	WP_193357670.1

Table S2 : Analysis of mutations in *qnr* gene in *E. bugandensis* 9i genome sequence

Mismatchs and positions	Position	Accession number of similar proteins with similar mismatches
G—S	P7	WP_218307411.1
A-D	P94	NA
A-V	P99	(GJK31478.1)
		WP_039992288.1

Table S3: Primers of the target β-lactamase genes

β-lactamases	Primer sequence (5′-3′)	Size (pb)	Reference
TEM	F :ATTCTTGAAGACGAAAGGGC	1150 bp	[42]
	R :ACGCTCAGTGGAACGAAAAC		
SHV	F :CACTCAAGGATGTATTG TG	885	
	R:TTAGCGTTGCCAGTGCTCG		
OXA-1	F :ACACAATACATATCAACTTCGC	813	
	R :AGTG TGTTTAGAATGGTGATC		
CTX-M-1 group	F:GTTACAATGTGTGAGAAGCAG	1041	
	R:CCGTTTC CGCTATTACAAA		
CTX-M-8 group	F:TGATGAGACATCGCGTTAAG	875	
	R:TAACCGTCGGTG ACGATTTT		
CTX-M-9 group	F:GTG ACAAAGAGAGTGCAACGG	857	
	R:ATGATTCTCGCCGCTGA AGCC		
CTX-M-10	F:CC GCGCTACACTTTGTGGC	944	
	R:TTACAAACCGTTGGTGA CG		
CMY1,CMY-8 toCMY-11	F :GCTGCTCAAGGAGCACAGGAT<br>R :CACATTGACATAGGTGTGGTGC	520	[43]
CMY-2 to CMY-7	F :TGGCCAGAACTGACAGGCAAA	462	
	R :TTTCTCCTGAACGTGGCTGGC		
DHA-1, DHA-2	F :AACTTTCACAGGTGTGCTGGGT	405	
	R :CCGTACGCATACTGGCTTTGC		
ACT-1	F:TCGGTAAAGCCGATGTTGCGG	302	
	R:CTTCCACTGCGGCTGCCAGTT		
IMP	F : GGAATAGAGTGGCTTAAYTCTC	232	[44]
	R : GGTTTAAYAAAACAACCACC		
SPM	F: AAAATCTGGGTACGCAAACG	271	
	R : ACATTATCCGCTGGAACAGG		
VIM	F : GATGGTGTTTGGTCGCATA	390	
	R : CGAATGCGCAGCACCAG		
OXA-48	F : GCGTGGTTAAGGATGAACAC	438	

	R : CATCAAGTTCAACCCAACCG		
GIM	F: TCGACACACCTTGGTCTGAA	477	
	R: AACTTCCAACTTTGCCATGC		
SIM	F: TACAAGGGATTCGGCATCG	570	
	R: TAATGGCCTGTTCCCATGTG		
NDM	F : GGTTTGGCGATCTGGTTTTC	621	
	R : CGGAATGGCTCATCACGATC		
KPC	F: CGTCTAGTTCTGCTGTCTTG	798	
	R: CTTGTCATCCTTGTTAGGCG		
IMI1-3 /NMC	F: TGCGGTCGATTGGAGATAAA	399	[45]
	R: CGATTCTTGAAGCTTCTGCG		
SME1-3	F: ACTTTGATGGGAGGATTGGC	551	
	R: ACGAATTCGAGCATCACCAG		
GES1-9	F: GCTTCATTCACGCACTATT	323	
	R: CGATGCTAGAAACCGCTC		

Table S4: Primers of the target antimicrobial resistance genes.

Target gene(s) or region	Primer sequence (5′-3′)	Size (pb)	Reference
tetA	F:GTAATTCTGAGCACTGTCGC<br>R:CTGCCTGGACAACATTGCTT	937	[39]
tetB	F:CTCAGTATTCCAAGCCTTTG<br>R:CTAAGCACTTGTCTCCTGTT	416	
sul1	F:TGGTGACGGTGTTCGGCATTC<br>R:GCGAGGGTTTCCGAGAAGGTG	789	
sul2	F:CGGCATCGTCAACATAACC<br>R:GTGTGCGGATGAAGTCAG	722	
sul3	F: CATTCTAGAAAACAGTCGTAGTTCG<br>R:CATCTGCAGCTAACCTAGGGCTTTGGA	990	
Int1	F:GGGTCAAGGATCTGGATTTCG	483	
	R: ACATGGGTGTAAATCATCGTC		
aac(3)-II	F:ACTGTGATGGGATACGCGTC	237	

	R:CTCCGTCAGCGTTTCAGCTA	
aac(3)-IV	F:CTTCAGGATGGCAAGTTGGT<br>R:TCATCTCGTTCTCCGCTCAT	286
qnrA	F:AGAGGATTTCTCACGCCAGG<br>R:TGCCAGGCACAGATCTTGAC	580
qnrB	F:GATCGTGAAAGCCAGAAAGG<br>R:ACGATGCCTGGTAGTTGTCC	469
qnrS	F:GCAAGTTCATTGAACAGGGT<br>R:TCTAAACCGTCGAGTTCGGCG	550
qepA	F:GCAGGTCCAGCAGCGGGTAG<br>R:GGACATCTACGGCTTCTTCG	617
aac(6')-Ib	F:TTGCGATGCTCTATGAGTGGCTA<br>R:CTCGAATGCCTGGCGTGTTT	482

Table S5. ANI values of *E. bugandensis* 9i isolate with all genome sequences of *E. bugandensis* deposited in genbank (as of December 2022).

Genome assebmy	ANI(%)
GCF 001054435.1_ ASM105443v1	95.23
GCF_023750845.1_ASM2375084v1	95.20
GCA_023750905.1_ASM2375090v1	95.16
GCF_023374275.1_ASM2337427v1	95.15
GCF 023154875.1 ASM2315487v1	95.15
GCA 023752545.1 ASM2375254v1	95.12
GCA 023152755.1 ASM2315275v1	95.10
GCA_023751035.1_ASM2375103v1	95.09
GCA 021287345.1 ASM2128734v1	95.08
GCA 023151965.1 ASM2315196v1	95.08
GCA_023153675.1_ASM2315367v1	95.08
GCA_023153675.1_ASM2315367v1	95.08
GCA 000515295.1 ASM51529v1	95.06
GCF 000515295.1 ASM51529v1	95.06
GCA 023151735.1 ASM2315173v1	95.06
GCA 021650125.1 ASM2165012v1	95.05
GCA 023151725.1 ASM2315172v1	95.05
GCA_023153975.1_ASM2315397v1	95.05
GCA_023153975.1_ASM2315397v1	95.05
GCF 903970915.1 ASM90397091v1	95.05
GCF 903970835.1 ASM90397083v1	95.05
GCA 018597305.1 ASM1859730v1	95.04

GCA 021650055.1 ASM2165005v1	95.03
GCA 021650105.1 ASM2165010v1	95.03
GCA_023154415.1_ASM2315441v1	95.02
GCA_023154415.1_ASM2315441v1	95.02
GCA_023750585.1_ASM2375058v1	95.02
GCF 900075815.1 12082 4 48	95.02
GCF 023752545.1 ASM2375254v1	95.02
GCF_023750585.1_ASM2375058v1	95.02
GCF 023154915.1 ASM2315491v1	95.02
GCA 015278715.1 ASM1527871v1	95.01
GCA 018597025.1 ASM1859702v1	95.01
GCF_023750405.1_ASM2375040v1	95.01
GCA 015137655.1 ASM1513765v1	95.00
GCF 015137655.1 ASM1513765v1	95.00
GCA 021650195.1 ASM2165019v1	95.00
GCA 023151635.1 ASM2315163v1	95.00
GCF 900324475.1 EB-247	95.00
GCA_023153995.1_ASM2315399v1	94.99
GCA_023153995.1_ASM2315399v1	94.99
GCF_023750245.1_ASM2375024v1_genomic.fna	94.99
GCA 019012175.1 ASM1901217v1	94.98
GCA 020042625.1 ASM2004262v1	94.98
GCA 018455145.1 PDT000712937.2	94.98
GCA 021650155.1 ASM2165015v1	94.98
GCA_023154055.1_ASM2315405v1	94.98
GCA_023154055.1_ASM2315405v1	94.98
GCF 900078165.1 11983 7 53	94.98
GCA 015704655.1 ASM1570465v1	94.97
GCA 023151665.1 ASM2315166v1	94.97
GCA_023153835.1_ASM2315383v1	94.97
GCA_023153835.1_ASM2315383v1	94.97
GCA_023752225.1_ASM2375222v1	94.97
GCA 017315445.1 ASM1731544v1	94.96
GCA 018455045.1 PDT000712935.2	94.96
GCA 023152215.1 ASM2315221v1	94.96
GCA_023153645.1_ASM2315364v1	94.96
GCA_023153645.1_ASM2315364v1	94.96
GCA 018598145.1 ASM1859814v1	94.95
GCA 023151655.1 ASM2315165v1	94.95
GCA 020099255.1 ASM2009925v1	94.94
GCA 023151825.1 ASM2315182v1	94.94

GCA_023153455.1_ASM2315345v1	94.94
GCA_023153535.1_ASM2315353v1	94.94
GCA_023153455.1_ASM2315345v1	94.94
GCA_023153535.1_ASM2315353v1	94.94
GCA 019815185.1 ASM1981518v1	94.93
GCF 023155015.1 ASM2315501v1	94.93
GCA 019351665.1 ASM1935166v1	94.92
GCA 021286605.1 ASM2128660v1	94.92
GCA 023152065.1 ASM2315206v1	94.91
GCF 023154785.1 ASM2315478v1	94.91
GCA 008641915.1 ASM864191v1	94.90
GCA 020097235.1 ASM2009723v1	94.90
GCA 023152155.1 ASM2315215v1	94.90
GCF 025535875.1 ASM2553587v1	94.90
GCA 008642035.1 ASM864203v1	94.89
GCF 023154985.1 ASM2315498v1 genomic.fna	94.89
GCA 019046905.1 ASM1904690v1	94.88
GCA 020099235.1 ASM2009923v1	94.87
GCA 018165775.1 PDT001012905.1	94.87
GCA 023151985.1 ASM2315198v1	94.87
GCA 015278695.1 ASM1527869v1	94.86
GCA 001054505.1 ASM105450v1	94.85
GCF 001054505.1 ASM105450v1	94.85
GCA 013366475.1 ASM1336647v1	94.85
GCA 013366505.1 ASM1336650v1	94.85
GCA 009647485.1 ASM964748v1	94.84
GCA 013366535.1 ASM1336653v1	94.84
GCA 023152455.1 ASM2315245v1	94.84
GCA_023683925.1_PDT001324297.1	94.84
GCA 013366465.1 ASM1336646v1	94.83
GCA 015684375.1 PDT000538113.1	94.83
GCA_023688005.1_PDT001324324.1	94.83
GCA 013366435.1 ASM1336643v1	94.82
GCA 020673825.1 ASM2067382v1	94.82
GCA 023153335.1 ASM2315333v1	94.82
GCA 021416825.1 ASM2141682v1	94.82
GCA 021649885.1 ASM2164988v1	94.82
GCA_023153955.1_ASM2315395v1	94.82
GCA_023684465.1_PDT001323984.1	94.82
GCA 013403425.1 ASM1340342v1	94.81

GCA 023152675.1 ASM2315267v1	94.81
GCA_023583125.1_PDT001317070.1	94.81
GCA_023588065.1_PDT001317179.1	94.81
GCA 023153435.1 ASM2315343v1	94.80
GCA 013403455.1 ASM1340345v1	94.79
GCF 001022595.1 ASM102259v1	94.78
GCA 020093305.1 ASM2009330v1	94.78
GCA 023152415.1 ASM2315241v1	94.78
GCF 023751985.1 ASM2375198v1	94.77
GCA_023751375.1_ASM2375137v1	94.76
GCA 023060615.1 ASM2306061v1	94.75
GCF 900075565.1 12082 4 47	94.75
GCA 008641635.1 ASM864163v1	94.74
GCF 902161535.1 25426 7 289	94.71
GCA 000958885.1 ASM95888v1	94.70
GCA 021287275.1 ASM2128727v1	94.70
GCA 001054625.1 ASM105462v1	94.69
GCF 001054625.1 ASM105462v1	94.69
GCA 023152335.1 ASM2315233v1	94.69
GCA 023152785.1 ASM2315278v1	94.66
GCA 001023305.1 ASM102330v1	94.64
GCF 001023305.1 ASM102330v1	94.64
GCA 023151855.1 ASM2315185v1	94.64
GCF 025535675.1 ASM2553567v1	94.62
GCA 021368735.1 PDT001216428.1	94.59
GCA 019815105.1 ASM1981510v1	94.58
GCF 024706625.1 ASM2470662v1	94.58
GCA 000957065.1 ASM95706v1	94.56
GCA 021368855.1 PDT001216423.1	94.55
GCA 015684255.1 PDT000538163.1	94.50
GCF 023751375.1 ASM2375137v1	94.50
GCF 025536315.1 ASM2553631v1	94.49
GCA 008642095.1 ASM864209v1	94.47
GCF 025535395.1 ASM2553539v1	94.42
GCF 903936295.1 ASM IMP80	94.41
GCA 022553555.1 ASM2255355v1	94.35
GCA 010590825.1 ASM1059082v1	93.91
GCA 010597605.1 ASM1059760v1	92.43
GCA 003964645.1 ASM396464v1	91.04
GCA 001518395.1 ASM151839v1	90.96
GCF 003964255.1 ASM396425v1	90.40
GCA 002860705.1 ASM286070v1	90.23

GCA 002848105.1 ASM284810v1	89.89
GCA 008641395.1 ASM864139v1	89.58
GCA 008641475.1 ASM864147v1	89.51
GCA 008640905.1 ASM864090v1	89.39
GCA 008641595.1 ASM864159v1	89.38
GCA 008640875.1 ASM864087v1	89.32
GCA 008640915.1 ASM864091v1	89.23
GCA 001518315.1 ASM151831v1	88.89
GCA 004804395.1 ASM480439v1	88.85
GCA 001055645.1 ASM105564v1	88.73
GCA 003964825.1 ASM396482v1	88.69
GCA 001057695.1 ASM105769v1	88.34
GCA 002785865.1 ASM278586v1	87.98
GCA 003965205.1 ASM396520v1	87.96
GCA 001055805.1 ASM105580v1	87.90
GCA 004801095.1 ASM480109v1	87.72
GCA 001055835.1 ASM105583v1	87.65
GCA 003985275.1 ASM398527v1	87.33
GCA 004804375.1 ASM480437v1	86.26
GCF 001054625.1 ASM105462v1	86.13
GCA 003627555.1 ASM362755v1	85.97
GCF 002890715.1 ASM289071v1	85.88
GCA 002890765.1 ASM289076v1	85.83
GCA 001631115.1 ASM163111v1	85.66
GCF 001631115.1 ASM163111v1	85.66
GCF 002890725.1 ASM289072v1	85.65
GCF 002890755.1 ASM289075v1	85.65
